# Florida Arsenic Distribution Index: Quantifying the Distribution of Past and Present Arsenic Usage

**DOI:** 10.3390/ijerph16050744

**Published:** 2019-03-01

**Authors:** Maya Scott-Richardson, Marilyn O’Hara Ruiz, Rebecca L. Smith

**Affiliations:** Department of Pathobiology, College of Veterinary Medicine, University of Illinois, Urbana-Champaign, Urbana, IL 61802, USA; moruiz@illinois.edu (M.O.R.); rlsdvm@illinois.edu (R.L.S.);

**Keywords:** arsenic, anthropogenic sources, natural sources, agriculture, indexing, geographic information systems, Florida, environment

## Abstract

Arsenic is an abundant, highly toxic element that is a global health concern due to damage from acute and chronic exposure and the potential for high local concentrations in heavily populated areas. In Florida, arsenic has been used heavily in agricultural, commercial, and industrial applications for decades. While studies have identified and quantified the contributions of arsenic to the state, there are fewer studies that have attempted to index to identify spatial distribution patterns. The aim of this study was to develop representative indices that would identify and estimate the distribution of arsenic from historic and present usage for the state of Florida at the county-level. Eight variables are summarized and categorized into two different types of arsenic indices that represent the arsenic distribution from natural occurrence and anthropogenic practices in Florida. The anthropogenic index had distributions scores that ranged from 0.20 to 1.60 with a mean of 0.61 (SD = 0.34). The natural index had distribution scores that ranged from 1.00 to 3.00 and a mean of 1.47 (SD = 0.43). Our finding noticed comparability between high arsenic distributions mainly occur in counties located in the northwestern and southwestern regions in both the anthropogenic and natural indices with diverse arsenic sources contributions.

## 1. Introduction

Anthropogenic use of arsenic in Florida has been found to contribute to the environmental contamination of water sources and surface soil [1]. Historically, arsenic has been used extensively as a pesticide, insecticide, herbicide, and crop desiccant in the forms of arsenic trioxide, lead arsenate, calcium arsenate and copper (II) acetoarsenite. Lead arsenate was widely used as a pesticide for apple and cherry orchards when applied as a foliar spray as it adhered well to plant surfaces, so the effects were longer lasting [2]. Arsenic trioxide was used to create arsenical dips to kill ticks that carried the parasite responsible for cattle fever and other tick-borne diseases [3,4]. Organic arsenic-containing chemicals, such as the herbicides Monosodium methanearsonate have been used to maintain the attractiveness of golf courses and turf [5,6]. Solo-Gabriele et al (2003) report that arsenic has been used extensively and in many forms within the state of Florida for the purpose of manufacturing goods such as fossil fuels, glass, and batteries as well as food products like animal feed and seafood [7]. Other sources of arsenic chemicals include chromate copper arsenate (CCA) wood, both treated in state and imported from other states. These CCA-treated wood products could be found in many areas of the state including homes, decks, utility poles, parks, playgrounds, and wood treatment plants [8]. Phosphate mining also released arsenic for use in fertilizers in and out of state [9].

Naturally occurring arsenic has been found in sedimentary, igneous, and metamorphic rocks, primarily associated with sulfide minerals such as orpiment, realgar (AsS), and arsenopyrite [9]. When rocks and minerals weather, arsenic may mobilize as arsenic salt compounds that can accumulate in the soil and plants [8,9]. This leads to low concentrations of arsenic in various water sources as a by-product of rock weathering as well as physical and chemical processes that break down soil containing mineral arsenic, which then leaches into water [10].

Groundwater is usually the main source of arsenic contamination due to its high accumulation of arsenic from weathering parent rock material and proximity to arsenic-containing minerals and contaminated sites from anthropogenic use. Previous studies (e.g., Solo-Gabriele et al. 2003 and Missimer et al., 2018) have estimated the total amount of arsenic released into Florida from multiple natural and anthropogenic sources and its consequence on environmental and public health [7,11]. Yet, we have found no studies that examined and visualized the distribution of arsenic from past and present arsenic usage in Florida. The aim of this study was to create representative indices that would identify and estimate the spatial distribution of arsenic from historic and present usage at the county level in the state of Florida. These indices are the first step to identifying counties with higher arsenic concentrations that may have health implications to residents of these areas.

## 2. Materials and Methods 

### 2.1. Overview

The 67 counties in the state of Florida, United States are the focus of this analysis. Data were identified pertaining to eight arsenic sources and processed to provide comparable county-level values of arsenic distribution potential. The eight sources include cattle dipping vats, monosodium methanearsonate, disodium methanearsonate, lead arsenate, phosphate mining, groundwater, and surface soils. These eight variables are summarized and categorized into two different types of arsenic indices that represent the arsenic distribution from natural occurrence and anthropogenic practices in Florida. Index ranking, spatial data processing, and mapping analysis were performed using SPSS 24 (IBM Corp, Armonk, NY, USA) and ESRI ArcMap 10.4 (Redlands, CA, USA). 

### 2.2. Source Identification and Arsenic Calculations

#### 2.2.1. Cattle Dipping Vats

The Florida Department of Health (FOH) and Florida Department of Environmental Protection (FDEP) assembled and maintain a list of the facility names of cattle dipping vats (CDV) and the county in which they were located based on state livestock records and permits. The list comprised 3241 total vats [12]. The United States Department of Agriculture and Livestock Sanitation Board regulated the amount of arsenic in the final solution [13,14], set at 8 lbs (2.2 lbs is equivalent to 1 kg) per 500 gal (1 gal (US gal) is equivalent to 3.78 L). Cattle dipping vats typically held up to 2000 gallons of arsenic dip [15]. To estimate how many pounds of arsenic were associated with dipping vats solutions for each county, ($lb_{As}^{CDV}$, 1), the federally-regulated amount of arsenic trioxide in pounds was multiplied by its arsenic fraction and divided it by the 500 gallons needed to create the arsenical solution to get the pounds of arsenic per gallon. The total gallons needed to fill a cattle-dipping vat, 2000 gallons, was multiplied by the pound of arsenic per gallon. The resulting product was multiplied by the total number of vats in each county,$\;CDV_{County}$. The arsenic fractions associated with $lb_{As}$ were determined by dividing the molecular weights of arsenic trioxide (*MW_ArsenicTrioxide_*) by the molecular weight of arsenic (*MW_As_*).

(1) $$lb_{As}^{CDV}=\;\left(\left(\frac{(8\;lbs_{Arsenic\;Trioxide}\;x\;\left(\frac{MW_{Arsenic\;Trioxide}}{MW_{As}}\right))}{500\;gal}\right)x\;2000\;gal\right)\;x\;CDV_{county}$$

#### 2.2.2. Monosodium Methanearsonate (MSMA) and Disodium Methanearsonate (DSMA)

MSMA and DSMA, two forms of organic arsenical herbicides, were used for cotton, turf, and near industrial sites [16]. The use of these organic herbicides in Florida has been reported from the 1950s (DSMA) and the 1960s (MSMA) until 2009 and used mainly for weed control along cotton fields, golf courses, and highways [17]. To estimate the arsenic distribution potential from DSMA and MSMA usage on cotton and citrus, we extracted county-level data on peak acreage in citrus and cotton production per decade between 1960 to 2010 from the United States Department of Agriculture National Agricultural Statistics Service (USDA NASS) database. According to the Environmental Protection Agency (EPA), the recommended amount of active ingredients of MSMA and DSMA to be administered in cotton and citrus fields is 2.0 to 2.25 pounds of active ingredients per acres, respectively [18]. According to Solo-Gabriele et al., 2003, the mean concentration of MSMA and DSMA herbicides active ingredients were roughly 0.50% of MSMA and 0.24% of DSMA [7]. To calculate the total pounds of arsenic from MSMA and DSMA usage,$(lb_{As}^{MSMA}$, 2; $lb_{As}^{DSMA}$, 3), for each county, the EPA recommended rate of each herbicide was divided by the percent active ingredient to attain the pounds of MSMA and DSMA per acre, which was then multiplied by the arsenic fraction of MSMA and DSMA respectively. This number was then multiplied by the total number of acres associated with peak cotton production per county (*TA_cotton_*). The arsenic fractions were determined by dividing the molecular weights of MSMA and DSMA (*MW_MSMA_*) by the molecular weight of arsenic (*MW_As_*).

(2) $$lb_{As}^{MSMA}=\;\;(\left(\frac{\frac{lb_{a.i.}}{A}}{\%\;a.i.}\right)\;x\;\left(\frac{MW_{MSMA}}{MW_{As}}\right))x\;TA_{cotton}$$

(3) $$lb_{As}^{DSMA}=\;\;(\left(\frac{\frac{lb_{a.i.}}{A}}{\%\;a.i.}\right)\;x\;\left(\frac{MW_{DSMA}}{MW_{As}}\right))\;\;x\;TA_{cotton}$$

#### 2.2.3. Lead Arsenate

Lead arsenate was the most common form of inorganic arsenical insecticide used for citrus crops [16]. The peak use of lead arsenate in Florida has been reported from the 1930s to the 1940s [19]. According to the EPA, the recommended amount of active ingredients of lead arsenate to be administered to citrus fields is 1.7 pounds of active ingredients per acres. The common concentration of active ingredients was roughly 0.70% of lead arsenate [20]. To calculate the pounds of arsenic from lead arsenate use ($lb_{As}^{LA}$), the EPA recommended rate of lead arsenate was divided by the percent active ingredient to attain the pounds of lead arsenate per acres, which was then multiplied by the arsenic fraction associated with lead arsenate. The pounds of arsenic per acre were then multiplied the total number of acres associated with citrus production from the 1960s to the 1980s by county (*TA_citrus_*). The arsenic fractions were determined by dividing the molecular weights of lead arsenate (*MW_LeadArsenate_*) by the molecular weight of arsenic (*MW_As_*).

(4) $$lb_{As}^{LA}=\;\;(\left(\frac{\frac{lb_{a.i.}}{A}}{\%\;a.i.}\right)\;x\;\left(\frac{MW_{Lead\;Arsenate}}{MW_{As}}\right))\;x\;TA_{citrus}$$

#### 2.2.4. Phosphate Mining

Based on a digital map of phosphate mining sites and reclamation efforts in Florida [21], we determined that five counties participated in phosphate mining activities. Phosphate rocks in Florida contain an average concentration of 7 mg/kg of arsenic [22]. Many of the sites were established in the 1970s; their current operational status is either still active, permanently shut down, or unknown. We calculated the total amount of acres that were associated with phosphate mining practices by county using ArcGIS. Data regarding the total number of phosphate rocks (in metric tons) mined from the years of 1970 to 2000 within the state of Florida were obtained in a report by Solo-Gabrielle et al., 2003 [7]. To calculate the total pounds of arsenic from phosphate mining activities for each county ($lb_{As}^{PR}$), the total number of phosphate rocks mined in metric tons was converted into kg, $\sum^{}PR_{1970-2000}$ and multiplied by the mean concentration of arsenic found in phosphate rocks then converted into pounds. The total pounds of arsenic were divided by the total acreage of mining sites within each county, ($A_{total}$), to calculate the total pounds of arsenic per acre in all counties. Finally, the pounds of arsenic per acre was multiplied by the total phosphate mining area for each individual county ($A_{county}$) to calculate the pound of arsenic associated with phosphate rock mining by county.

(5) $$lb_{As}^{PR}=\;\left(\frac{\left(\;\sum^{}PR_{1970-2000}\left(kg\right)x\;7\frac{mg}{kg}\right)\;x\;10^{-6}\frac{kg}{mg}\;x\;\frac{2.204624\;lb}{1\;kg}}{A_{total}}\right)\;x\;A_{county}$$

#### 2.2.5. Background Groundwater Levels

Point-level well data was extracted using ArcGIS from the United States Geological Survey (USGS) database on arsenic in 585 groundwater samples from public and domestic water supplies, industrial, research, and agricultural wells in the state of Florida [23,24]. Inverse distance weighted interpolation was used to estimate the arsenic levels across the state, depicted as a GIS raster grid, from the arsenic level data points. For each county, the mean of the arsenic estimates was calculated using zonal statistics. Results are reported in parts per million (ppm).

#### 2.2.6. Background Surface Soil Levels

Similarly, 89 samples from the soil surface layer (Organic Matter, or O horizon, if present) across Florida between 2007 to 2010 have been tested to estimate arsenic concentrations as part of the North American Soil Geochemical Landscapes project [25]. Inverse distance weighted interpolation was used to estimate the arsenic levels across the state for each of the three horizon levels as GIS raster grids based on the arsenic levels from the sample points in Florida. The mean of the arsenic estimates was calculated for each county using zonal statistics. Results are reported in ppm.

### 2.3. Indexing Approach

The sources were classified into either natural or anthropogenic group based on their primary or secondary source and usage. For this study, two types of indices were created: a natural source arsenic distribution index and an anthropogenic source arsenic distribution index. Using SPSS, these counties were given a distribution score (DS) between 1 and 3, representing low to high distribution. The distribution scores were created by identifying the natural breaks by grouping each individual anthropogenic and natural source with corresponding pounds of arsenic or arsenic concentrations, respectively by counties (Figure S1). In cases where a specific arsenic source did not occur in a county, the county received a distribution score of zero (0) to represent no distribution from that source. To create the Florida arsenic distribution indices (FADI), the individual arsenic sources pertaining to either anthropogenic or natural sources were averaged, resulting in a final distribution source that ranges from 0 to 3, representing absence to high distribution. Dataset is available as supplementary material (FADI Dataset).

(6) $$FADI_{Anthropogenic}=\;\left(\frac{DS_{CDV}x\;DS_{MSMS}\;x\;DS_{DSMA}\;x\;DS_{LA}\;x\;DS_{PR}}{5}\right)$$

(7) $$FADI_{Natural}=\;\left(\frac{DS_{GW}x\;DS_{Topsoil}}{2}\right)$$

## 3. Results and Discussion

**Figure 1:**
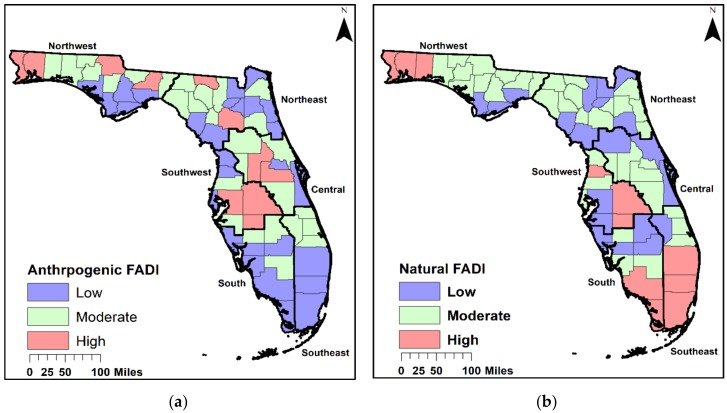
The Florida Arsenic Distribution Indices (FADIs): (**a**) anthropogenic source FADI; (**b**) natural source FADI.

For the anthropogenic FADI, values ranged from 0.20 to 1.60 with a mean of 0.61 (SD = 0.34). The anthropogenic FADI had a group of 28 counties within the low arsenic distribution (the minimum distributions from contributing sources), 28 counties considered moderate (the averaged distributions from contributing factors), and 11 counties with higher arsenic distribution (the highest distributions from contributing sources), comparatively (Figure 1a). The natural FADI had values that ranged from 1.00 to 3.00 and a mean of 1.47 (SD = 0.43). The natural FADI had a group of 20 counties within the low arsenic distribution, 36 counties considered moderate, and 11 counties within the higher arsenic distribution category (Figure 1b). These indices were created with the specific purpose of quantifying comparable means of past arsenic distribution from various sources that can be used for understanding the use of arsenic in Florida.

The counties with the lowest arsenic distribution from anthropogenic practices were in the Northeast, northwestern, southeastern, and the southern regions of Florida. In the southern regions, fewer cattle dipping vats (CDVs) have been identified meaning that less arsenic is distributed from this source. No mining activities took place in these areas except for one county in the northeastern region (Hamilton). Since cotton was grown mainly in certain northern counties, MSMA and DSMA were not commonly used in the Southern region leading to the absence or low arsenic distribution from these arsenicals. Lead arsenate was rarely used in northern counties because citrus production took place mainly in the central and southern regions. Thus, leading to minimal (none reported to low) arsenic distribution of lead arsenate in northern counties. The regions with the highest anthropogenic arsenic distribution were in the northwestern part of the state outside the panhandle and the Southwest. In the northwestern region, these counties have more arsenic distribution from agricultural practices such as CDVs, MSMA, and DSMA usage. Counties in the southwestern region have more CDVs, lead arsenate, and phosphate mining activities and practices.

The highest arsenic distributions from natural sources were in the northwestern, southwestern, and southeastern counties near Miami. Counties identified as high in the northwestern region were found to have moderate to high groundwater arsenic distribution. Counties in the southern and southeastern regions had low to moderate arsenic distribution while surface soils were identified as having an arsenic distribution that was moderate to high. Northeastern and central regions had no high arsenic distribution. Counties in these regions had low to moderate arsenic distribution in groundwater and surface soils. This pattern was noticed in other regions as well. Comparing the arsenic distribution of the anthropogenic arsenic sources to natural arsenic sources, we find similarities of high distribution mainly occurring in counties located in the northwestern and southwestern regions. These counties had moderate to high arsenic distributions in most sources with the exception being groundwater. Surface soils arsenic distributions in many of these areas were found to be moderate to high which may be related to the moderate to high arsenic distributions found from agricultural activities such as CDVs, arsenicals, and phosphate mining practices.

We recognize several limitations to our study. There are many anthropogenic sources of arsenic that have not been accounted for in the indices. In the case of cattle dipping vats, many vats are unaccounted for due to the deconstruction of vats or misplacement of records pertaining to vat locations. According to the Florida Department of Health (FDOH) and the FDEP, there are approximately 3400 cattle dipping vats located in the state of Florida; yet we only have records for 3241 vats [26,27]. This means that the database used to create our dipping vat arsenic contribution can be updated as more cattle dipping vats are discovered. Other arsenical chemicals used in agricultural practices also were not included in our anthropogenic index. We chose to include the most commonly used and well-documented inorganic and organic arsenic-containing chemical used for agricultural practices—MSMA, DSMA, and lead arsenate. In the future, we hope to include other arsenicals such as calcium arsenate, sodium arsenite, copper (II) acetate triarsenite (Paris Green), and roxarsone (in chicken feed and litter) when more information, including the amount of solution used and the years used, become available.

Chromated Copper Arsenate (CCA) treated wood was not included in this index. Different types of CCA-treated wood have been used for the construction of residential properties, commercial properties, gardening and farming spaces, and parks/recreational areas [28]. The use of CCA-treated wood was clearly widespread in Florida, but the actual number of land parcels affected by the arsenic-containing compound was not recorded. Studies that evaluate the extent of arsenic release from CCA-treated wood are limited; however, Khan et al., 2006a found inorganic arsenic (III) and arsenic (V) in runoff and infiltrated water below CCA-treated decks [29]. After disposal of CCA-treated wood in landfills, the arsenic can leach into the disposal site which can affect groundwater arsenic concentrations [30,31]. Incineration of CCA-treated wood can also cause arsenic and the other chemicals to be admitted into the air along with the ash from the burnt wood which can leach into the environment and be breathed in by humans as a particulate [28]. However, quantification of the use of CCA across Florida proved to be beyond the scope of this study. When assessing natural sources of arsenic from surface soil and groundwater, we were unable to separate the natural baseline concentrations of arsenic and the added concentrations from anthropogenic activities. Partitioning these differences in concentrations from the natural sources is complicated due to only secondary data being available. Our indices can be modified in the future to include input from quantification of such other sources of arsenic.

## 4. Conclusions

The FADIs that we developed can help raise awareness of the complex overlapping spatial patterns of arsenic in Florida. The indices present comparable quantifications for arsenic with combined arsenic distributions from multiple sources within each county. Our findings show that high arsenic distributions are found in counties residing in the northern and southern regions of Florida with diverse arsenic sources contributions. These county-level arsenic indices are an important step to developing spatial risk analyses at a scale that can be used to better identify and prioritize areas of the population-level risk of arsenic exposure. These broad-scale characterizations of arsenic across the state must be followed by more thorough studies at both the local and community levels in Florida. More detailed investigations of arsenic and evaluation of local human exposure will be useful to investigate both acute and chronic arsenic exposure.

## Supplementary Materials

The following are available online at https://www.mdpi.com/1660-4601/16/5/744/s1, Figure S1: The frequency of individual arsenic scored using the Florida Arsenic Distribution indexing approach. Dataset: FADI Dataset.
