# Erratum: Florida Arsenic Distribution Index: Quantifying the Distribution of Past and Present Arsenic Usage

**DOI:** 10.3390/ijerph17010270

**Published:** 2019-12-30

**Authors:** Maya Scott-Richardson, Marilyn O’Hara Ruiz, Rebecca L. Smith

**Affiliations:** Department of Pathobiology, College of Veterinary Medicine, University of Illinois, Urbana-Champaign, Urbana, IL 61802, USA; moruiz@illinois.edu (M.O.R.);

The authors wish to add the following correction to their paper published in IJERPH [1]. In the Materials and Methods section under the *Indexing Approach* Section 2.3, the authors wish to replace

(6) $$FADI_{Anthropogenic}=\left(\frac{DS_{CDV}x\;DS_{MSMS}\;x\;DS_{DSMA}\;x\;DS_{LA}\;x\;DS_{PR}}{5}\right)$$

(7) $$FADI_{Natural}=\left(\frac{DS_{GW}x\;DS_{Topsoil}}{2}\right)$$

with

(6) $$FADI_{Anthropogenic}=\left(\frac{DS_{CDV}+DS_{MSMA}+DS_{DSMA}+DS_{LA}+DS_{PR}}{5}\right)$$

(7) $$FADI_{Natural}=\left(\frac{DS_{GW}+DS_{Topsoil}}{2}\right)$$

The authors would like to apologize for any inconvenience caused to the readers. The change does not affect the results of this study.
