# Supplementary material for: Florida Arsenic Distribution Index: Quantifying the Distribution of Past and Present Arsenic Usage
Source: Int J Environ Res Public Health. 2019 Mar 1;16(5):744. doi: 10.3390/ijerph16050744 (PMC6427290; doi:10.3390/ijerph16050744)
Supplement: Supplementary file 1 [file ijerph-16-00744-s001.zip › ijerph-436434-supplementary.pdf]

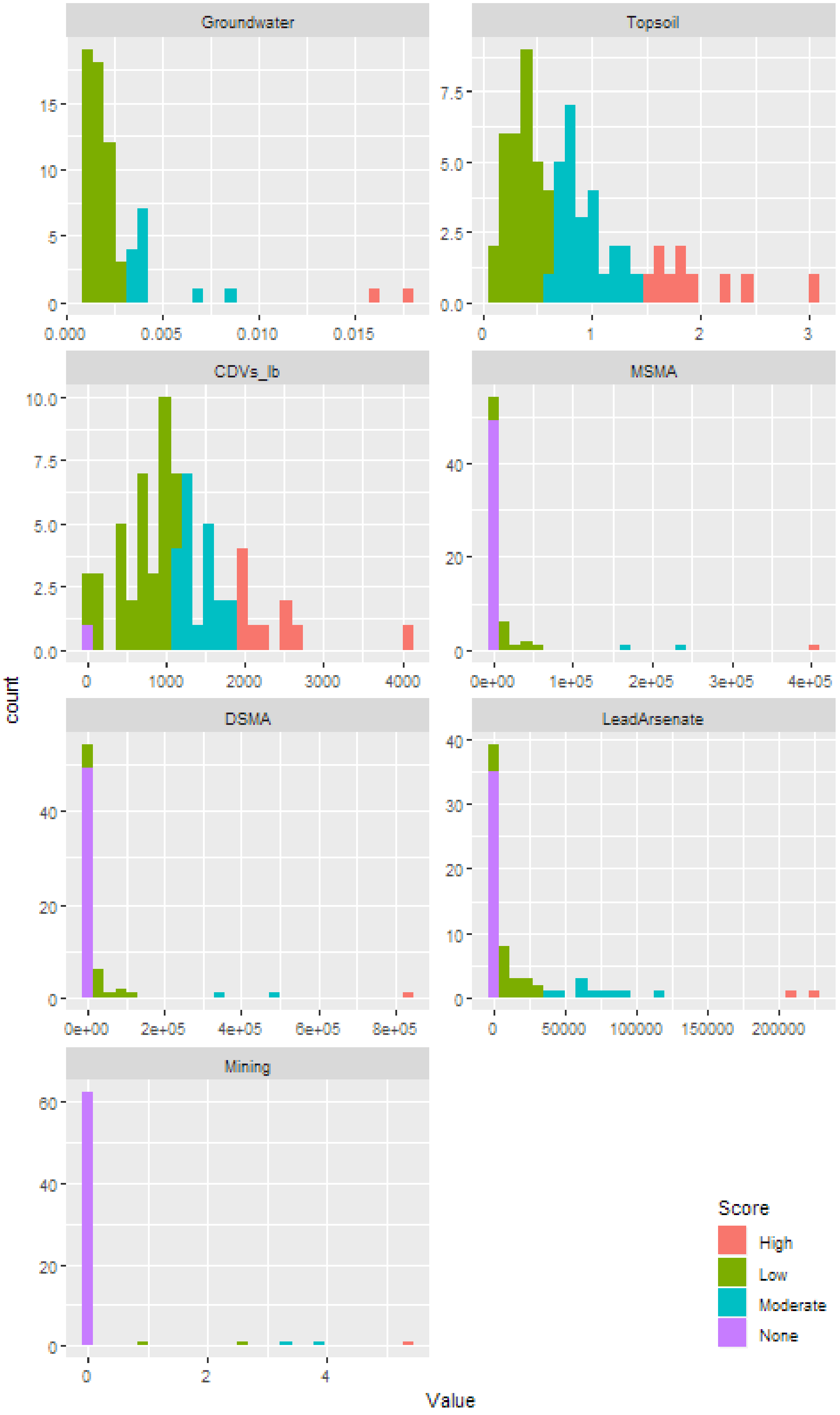

Figure 1. The frequency of individual arsenic scored using the Florida Arsenic Distribution indexing approach.
